# *Planococcus maritimu* ML1206 Strain Enhances Stress Resistance and Extends the Lifespan in *Caenorhabditis elegans* via FOXO/DAF-16

**DOI:** 10.3390/md21010001

**Published:** 2022-12-20

**Authors:** Jing-Shan Wu, Chun-Guo Lin, Chang-Long Jin, Yan-Xia Zhou, Ying-Xiu Li

**Affiliations:** 1 Marine College, Shandong University, Weihai 264209, China; 2 School of Mechanical, Electrical and Information Engineering, Shandong University, Weihai 264209, China

**Keywords:** probiotics, *Caenorhabditis elegans* (*C. elegans*), longevity, oxidative stress, *Planococcus maritimu*

## Abstract

The antioxidant effect of probiotics has been widely recognized across the world, which is of great significance in food, medicine, and aquaculture. There are abundant marine microbial resources in the ocean, which provide a new space for humans to explore new probiotics. Previously, we reported on the anti-infective effects of *Planococcus maritimu* ML1206, a potential marine probiotic. The antioxidant activity of ML1206 in *C. elegans* was studied in this paper. The study showed that ML1206 could improve the ability of nematodes to resist oxidative stress and effectively prolong their lifespan. The results confirmed that ML1206 could significantly increase the activities of CAT and GSH-PX, and reduce the accumulation of reactive oxygen species (ROS) in nematodes under oxidative stress conditions. In addition, ML1206 promoted DAF-16 transfer to the nucleus and upregulated the expression of *sod-3*, *hsp-16.2*, and *ctl-2*, which are downstream antioxidant-related genes of DAF-16. Furthermore, the expression of the SOD-3::GFP and HSP-16.2::GFP was significantly higher in the transgenic strains fed with ML1206 than that in the control group fed with OP50, with or without stress. In summary, these findings suggest that ML1206 is a novel marine probiotic with an antioxidant function that stimulates nematodes to improve their defense abilities against oxidative stress and prolong the lifespan by regulating the translocation of FOXO/DAF-16. Therefore, ML1206 may be explored as a potential dietary supplement in aquaculture and for anti-aging and antioxidant purposes.

## 1. Introduction

Numerous studies have shown that resistance to oxidative stress is crucial to maintaining health and extending the lifespan of organism. Under the normal physiological state of an organism, ROS, which refers to oxygen-centered free radicals and their metabolites, is in a dynamic balance between generation and elimination [1,2]. The free radical theory proposes that if oxygen free radical formation is uncontrolled and the balance of antioxidant protection is broken, it will lead to the decline of immune ability and an increased incidence of oxidative stress-related diseases in creatures [3].

Finding new antioxidant compounds that can reduce oxidative stress is important. Probiotics have been officially defined as “live microorganisms which, when administered in adequate amounts, confer a health benefit on the host” [4]. Probiotics have been widely used in the fields of medicine, food, and aquaculture due to their excellent probiotic functions, such as antibacterial, immunity enhancement, and antioxidant activity [5]. They mainly come from the gastrointestinal tract of host animals or their living environment [6]. There are abundant microbial resources in the ocean, which provides a broad space for humans to explore probiotics. To date, the potential probiotics isolated from the marine environment have mainly included yeast, *Bacteroidetes*, *actinobacteria*, and *firmicutes* [7]. These microorganisms could play a beneficial role on the host by scavenging reactive oxygen radicals, activating antioxidant enzyme systems, and activating endogenous antioxidant signaling pathways [8]. However, more in-depth studies are needed to elucidate the underlying molecular mechanisms of probiotics. *Planococcus maritimu* ML1206 (*P. maritimu*) is a potential marine probiotic isolated from the intestines of oysters and sea bass. It has strong antibacterial effects, both acid and bile salt resistance, safe and non-toxic properties. In addition, ML1206 can inhibit the intestinal colonization of pathogens without affecting the normal physiological indexes of nematodes [9]. However, the biological activity of *P. maritimu* ML1206 remains to be studied. 

*Caenorhabditis elegans* (*C. elegans*) is considered to be one of the best model organisms for the study of antioxidant activity and underlying mechanisms [10]. *C*. *elegans* is a multicellular model organism, which has the advantages of small size, low cost, simple anatomy, transparent structure, easiness of culture, short growth cycle, and a fully known genome sequence; it is also easily modified under genetic manipulation [11,12]. In addition, their behavioral and physiological indicators, such as swallowing rate, lipofuscin accumulation, and exercise, can reflect the aging rate and health status to some extent, and thus contribute to the study of the effects of different metabolites on oxidative damage and longevity [13,14]. In *C. elegans*, the *DAF-2*/*DAF-16* pathway (insulin-like signaling pathway), p38-MAPK pathway (mitogen-activated protein kinase pathway) and DBL-1/TGF-β pathway are important signaling pathways affecting the longevity and oxidative stress response in *C. elegans*. When *DAF-16* is dephosphorylated, it is transferred into the nucleus, thereby activating the transcriptional expression of antioxidant genes [15]. Hisako et al. reported the beneficial effects of *Lactobacillus gasseri* SBT2055 (LG2055) on nematode longevity due to their ability to enhance resistance to oxidative stress and stimulate the innate immune response [16]. Some studies have also shown that probiotics (such as *Lactobacillus*, *Bifidobacterium*, and *Enterococcus*) improve the resistance of nematodes to oxidative stress by reducing the accumulation of exogenous ROS [17,18,19]. 

When the body is infected, it will release a great deal of ROS to destroy the cell structure and cause oxidative stress damage [20]. Therefore, anti-infection and anti-oxidation activities are closely related. The previous study has demonstrated that ML1206 could activate the innate immune response and protect nematodes from *V. anguillarum* infection [9]. Based on this, the antioxidant effects of ML1206 in *C. elegans* were discussed in this study. The effect of feeding ML1206 on the lifespan of *C. elegans* under normal or oxidative stress conditions was investigated. In addition, the survival effects of feeding ML1206 on *C. elegans* under different environment stress were investigated. Then, several kinds of antioxidant enzyme activity and the level of ROS were determined. Furthermore, the antioxidant mechanism of ML1206 was elucidated in *C. elegans* using the real-time quantitative PCR technique, and transgenic strains expressing fluorescence-reported genes and mutants. In summary, the results showed that ML1206, as a benefit probiotic, could protect nematodes against oxidative stress, and have the potential to improve host longevity.

## 2. Results

### 2.1. Effect of ML1206 on the Lifespan of C. elegans

**Figure 1:**
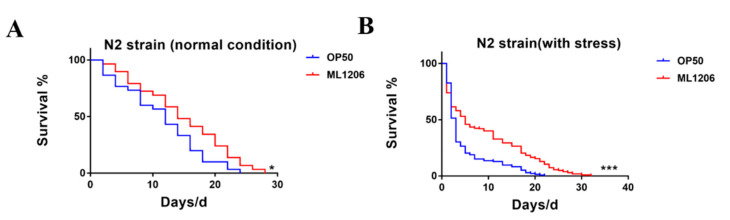
Effect of ML1206 on the lifespan of *C. elegans*: (**A**) survival curve of N2 nematodes fed with OP50 or ML1206 under normal condition; and (**B**) survival curve of N2 nematodes fed with OP50 or ML1206 exposed to oxidative stress induced by H_2_O_2_. The numbers of nematodes are calculated in Kaplan-Meier diagrams, and statistical significance was evaluated by the log-rank test. *p* < 0.05 is considered statistically significant. (*: *p* < 0.05; ***: *p* < 0.001).

We evaluated the effect of ML1206 on the lifespan of N2 nematodes. It was observed that the nematodes fed with ML1206 showed a longer lifespan (Figure 1A), which represented an increase in the mean lifespan, from 11.47 ± 1.18 days to 14.76 ± 1.35 days, of 28.7% compared with the control group fed with OP50 (Table 1). The increase of lifespan had a good correlation with oxidative stress resistance [21]. Based on this, the entire lifespan of nematodes fed with ML1206 or OP50 was determined under H_2_O_2_-induced oxidative stress. As shown in Figure 1B, the feeding of ML1206 markedly increased the mean lifespan of N2 nematodes by 48.8%, from 4.63 ± 0.44 days to 9.04 ± 0.61 days under oxidative stress, compared with the control group fed with OP50 (Table 1). The results suggested that ML1206 could extend the lifespan of *C. elegans* with or without oxidative stress, and exhibited a substantial protective effect against oxidative stress.

### 2.2. Effect of ML1206 on the Oxidative Stress Resistance of C. elegans

**Figure 2:**
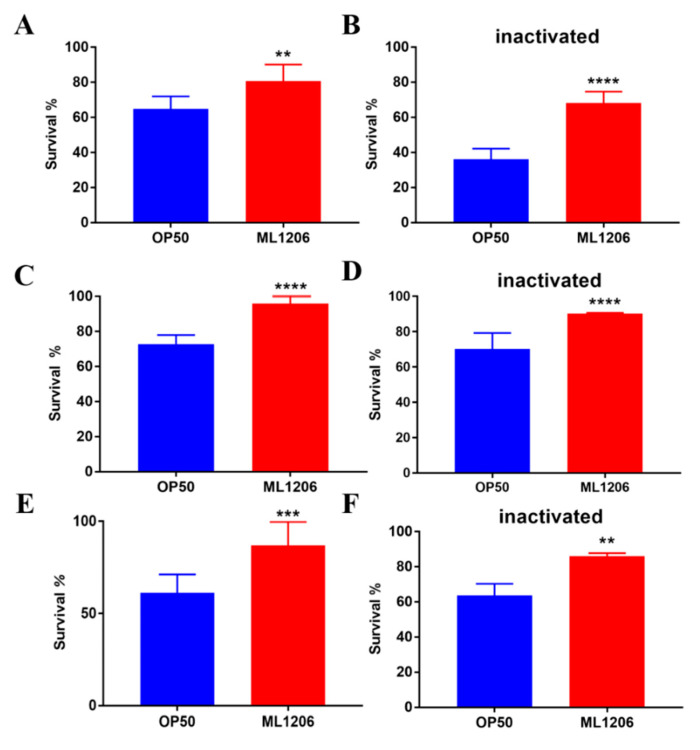
Effect of ML1206 on the oxidative and heat stress in *C. elegans*: (**A**) survival of nematodes fed with live OP50 and ML1206 under PQ-induced oxidative stress; (**B**) survival of nematodes fed with inactivated OP50 and ML1206 under PQ induced oxidative stress; (**C**) survival of nematodes fed with live OP50 and ML1206 under H_2_O_2_ induced oxidative stress; (**D**) survival of nematodes fed with inactivatedOP50 and ML1206 under H_2_O_2_ induced oxidative stress; (**E**) survival of nematodes fed with live OP50 and ML1206 under heat stress; and (**F**) survival of nematodes fed with inactivated OP50 and ML1206 under heat stress. All exposures were performed in nematodes after ML1206 or OP50 feeding for 48 h. Results are represented as mean ± SEM of three independent experiments and were statistically analyzed by an unpaired Student’s *t*-test. (**: *p* < 0.01; ***: *p* < 0.001; ****: *p* < 0.0001).

Next, we investigated whether feeding ML1206 could improve the resistance to oxidative stress of nematodes. Hydrogen peroxide and Parquat (PQ) are the most commonly employed chemical stressors [22]. We assessed the resistance to oxidative stress induced by PQ and H_2_O_2_ of *C. elegans* treated with ML1206, by comparing the survival rate of nematodes fed with either live or inactivated OP50 and ML1206. In the PQ-induced stress model, Figure 2A shows that the survival rate of nematodes fed with live ML1206 increased by 24.32% compared with the control group fed with OP50; Figure 2B shows that compared with nematodes fed with inactivated OP50, the survival rate of nematodes fed with inactivated ML1206 further increased by 88.92%. Similarly, in the H_2_O_2_-induced oxidative injury model, the survival rate of nematodes fed with live ML1206 was increased by 31.75% compared with the group fed with live OP50 (Figure 2C). Also, the results showed a significant increase of 28.57% of survival in the nematodes fed with inactivated ML1206 in comparison with the control group fed with inactivated OP50 (Figure 2D). The results showed that feeding both live and inactivated ML1206 significantly enhanced the resistance to oxidative stress of *C. elegans*.

### 2.3. Effect of ML1206 on the Heat Stress Resistance of C. elegans

In order to investigate whether feeding ML1206 could improve the heat stress resistance of nematodes, the survival of nematodes fed with live or inactivated OP50 and ML1206 was observed. Compared with the control group fed with OP50, the survival of *C. elegans* fed with ML1206 increased by 41.93% (Figure 2E). Figure 2F showed that nematodes fed with inactivated ML1206 had a significant increase of 35.07% in survival rate compared to the control group fed with inactivated OP50. The results showed that feeding both live and inactivated ML1206 significantly improved the survival of nematodes under heat-stress conditions increased via the enhancement of stress resistance in *C. elegans*. In addition, there was no significant difference of the survival between the groups fed with ML1206 or OP50 in the presence of Ultraviolet (UV) stress (Figure S1), indicating that ML1206 couldn’t protect *C. elegans* from UV stress. 

### 2.4. Effect of ML1206 on the Antioxidant Activity in C. elegans

**Figure 3:**
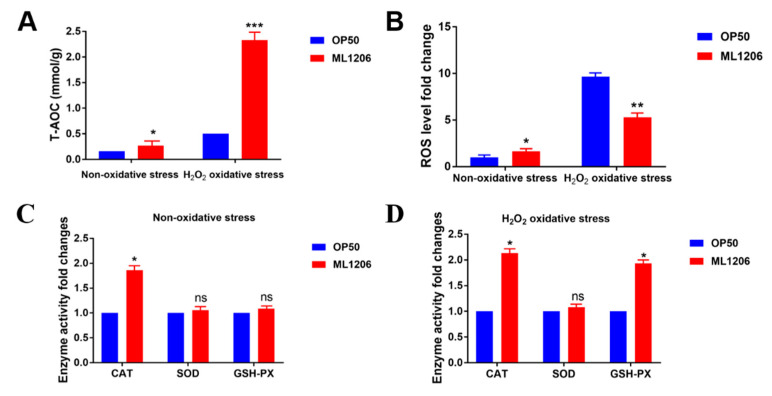
Effects of ML1206 on the antioxidant capacity in *C. elegans*: (**A**) the effect of ML1206 on the T-AOC of nematodes with or without stress; (**B**) effect of ML1206 on ROS level in the presence or absence of oxidative stress in *C. elegans*; (**C**) the effect of ML1206 on the enzyme activities of nematodes in the absence of oxidative stress; and (**D**) the effect of ML1206 on the enzyme activity in nematodes. Results are represented as mean ± SEM of three independent experiments was statistically analyzed by unpaired Student’s *t*-test. *p* < 0.05 is considered statistically significant. (*: *p* < 0.05; **: *p* < 0.01; ***: *p* < 0.001, ns: *p* > 0.05).

The FRAP assay is a method to measure the reduction ability of ferric iron in plasma and tissues and is often used to evaluate the total antioxidant ability (T-AOC) [23]. The activities of antioxidant enzymes, for example, Superoxide Dismutase (SOD), Catalase (CAT), and Glutathione peroxidase (GSH-PX), are also commonly used indicators for evaluating antioxidant capacity [24]. ML1206 strain have no obvious T-AOC activity and ability to scavenge free radicals *in vitro* (Figures S2 and S3), but ML1206 showed well antioxidant activity in *C. elegans* and enhanced oxidative stress resistance of them. Here, to further explore how ML1206 enhance resistance to oxidative stress of *C. elegans*, we detected the T-AOC, CAT, SOD, and GSH-PX activities of nematodes fed with OP50 and ML1206 with or without oxidative stress. The results showed that the T-AOC of nematodes fed with ML1206 was significantly increased to 1.6 fold in the absence of stress (Figure 3A). Also, it was also shown that the T-AOC in *C. elegans* fed with ML1206 was enhanced compared with the control group fed with OP50 in the presence of stress, suggested by a 3.6-fold increase. The CAT enzyme activity of the nematodes fed with ML1206 was significantly higher than that of the control under non-oxidative stress (Figure 3C). The CAT and GSH-PX activities of the nematodes fed with ML1206 were significantly higher than those of the control (Figure 3D). In conclusion, the enhanced antioxidant capacity of nematodes fed with ML1206 may be related to the increase of the antioxidant enzymes’ activity.

### 2.5. Effect of ML1206 on ROS Accumulation in C. elegans

Excessive ROS could destroy the redox homeostasis in the cell and even promote cell apoptosis [25]. In order to explore the reason why ML1206 enhances the resistance to oxidative stress in *C. elegans*, synchronized nematodes were covered with or without 5 mM H_2_O_2_ and exposed to the superoxide probe H_2_DCF-DA to evaluate the influence of ML1206 on the ROS accumulation in *C. elegans*. As shown in Figure 3B, the ROS level of nematodes fed with ML1206 and OP50 was similar under nomal conditions; while the ROS level of *C. elegans* fed with ML1206 significantly decreased by 53.7% compared with that of the control group fed with OP50 under oxidative stress. We concluded that feeding ML1206 exhibited a strong reduction of ROS in *C. elegans* under oxidative stress conditions.

### 2.6. Effect of ML1206 on the Antioxidant Genes Expression in C. elegans

**Figure 4:**
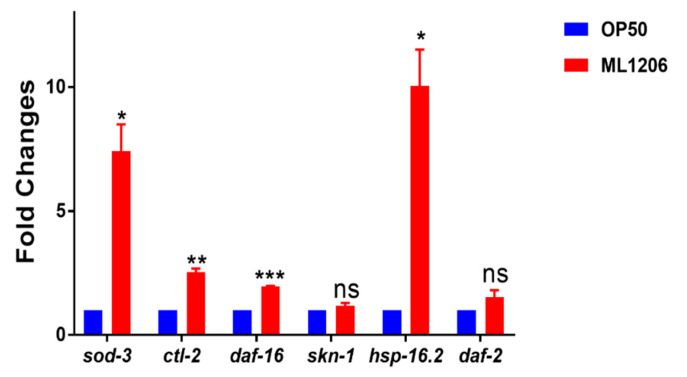
Effect of ML1206 on the relative expression of the antioxidant-related gene in *C. elegans*. The mRNA level of genes was quantified by the real-time PCR, which was normalized with that of *act-1* as a control; *p* < 0.05 is considered statistically significant. (ns: No significant difference, *: *p* < 0.05; **: *p* < 0.01; ***: *p* < 0.001).

The mRNA level of the genes related to antioxidants can reflect the antioxidant capacity of nematodes to a certain extent. Here, the effect of ML1206 on the expression of several antioxidant genes, including *sod-3*, *ctl-2*, DAF-16, *hsp-16.2*, *skn-1* and DAF-2, was studied. The results showed that the expression of *sod-3*, *ctl-2*, DAF-16, and *hsp-16.2* were significantly upregulated by 6.4-, 1.54-, 1-, and 9-fold, respectively, in *C. elegans* fed with ML1206 compared with the control group fed with OP50, while no significant difference was observed for *skn-1* and DAF*-2* genes between the experimental group and control group (Figure 4). Our results provides evidence from the mRNA level that ML1206 could improve the antioxidant capacity in *C. elegans* by regulating certain oxidative stress-related genes.

### 2.7. Effect of ML1206 on Nuclear Translocation of DAF-16 in C. elegans

**Figure 5:**
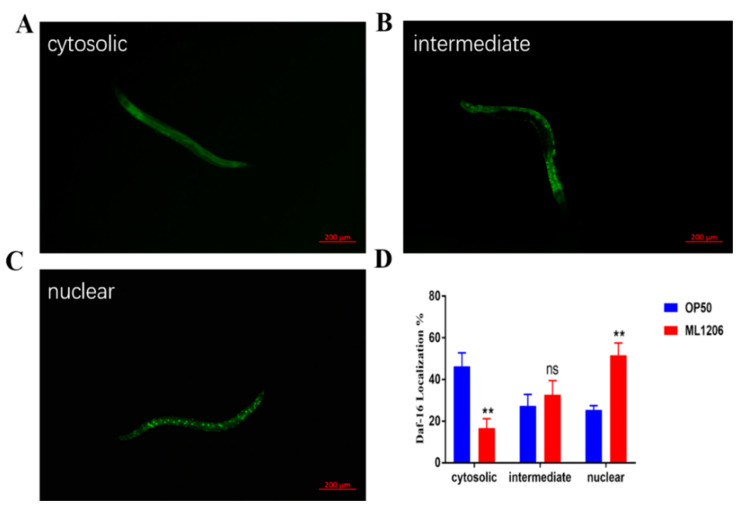
Effect of ML1206 on the DAF-16::GFP nuclear location in *C. elegans*: (**A**) representative images of the DAF-16::GFP location mainly in the cytoplasm of *C. elegans*; (**B**) representative images of the DAF-16::GFP location in the cytoplasm and nucleus of *C. elegans*; (**C**) representative images of the DAF-16::GFP location mainly in the nucleus of *C. elegans*; and (**D**) DAF-16::GFP localization in different areas of nematodes fed with OP50 and ML1206. The results are represented as mean ± SEM from three repeated experiments with 10 nematodes in each group, and the statistical significance is evaluated by Student’s *t*-test. (**: *p* < 0.01; ns: *p* > 0.05). Scale bar = 200 µm.

DAF-16 is a transcription factor (FOXO) that plays an important role in oxidative stress and aging in *Caenorhabditis elegans* and mammals [26]. To examine the role of DAF-16 in nematodes fed with ML1206, the nuclear translocation of DAF-16::Green fluorescent protein (GFP) was investigated. Figure 5A showed that DAF-16 was normally localized in the cytosol, and when activated, it is found that DAF-16::GFP of nematodes fed with ML1206 was mainly concentrated in the nucleus compared with the control group fed with OP50 (Figure 5C). The relative localization frequency of DAF-16::GFP in the nuclear of nematodes fed with ML1206 was significantly increased by 26.34, from 25.33% to 51.67% (Figure 5D). The results showed that ML1206 could activate DAF-16 and promote its nuclear translocation in *C. elegans*.

### 2.8. Effect of ML1206 on the sod-3::GFP and hsp-16.2::GFP Expressions in C. elegans

**Figure 6:**
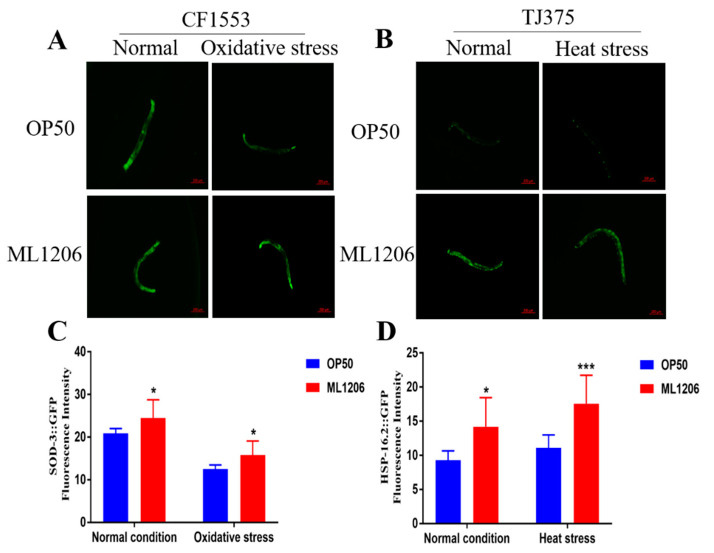
Representative images of the SOD-3::GFP and HSP-16.2::GFP expression of transgenic strains in the presence or absence of stress: (**A**) representative images of the SOD-3::GFP expression in the presence or absence of oxidative stress of transgenic strain CF1553; (**B**) representative images of the HSP-16.2::GFP expression in the presence or absence of heat stress of transgenic strain TJ375; (**C**) relative fluorescence intensity of OP50 and ML1206 group in the presence or absence of oxidative stress in transgenic strain CF1553; and (**D**) relative fluorescence intensity of OP50 and ML1206 group in the presence or absence of heat stress in transgenic strain TJ375. The results are represented as mean ± SEM from three repeated experiments with 10 nematodes in each group, and the statistical significance is evaluated by Student’s *t*-test. (*: *p* < 0.05; ***: *p* < 0.001). Scale bar = 200 µm.

*Sod-3* is an important regulator of longevity and stress resistance in *C. elegans*, and *Sod-3* expression indirectly indicates the DAF-16 transcriptional activity [27]. We used transgenic strain CF1553 to quantify the expression level of SOD-3::GFP. In the absence of stress, the relative fluorescence intensity of SOD-3::GFP of nematodes fed with ML1206 increased by 18% compared with the nematodes fed with OP50. The relative fluorescence intensity of the nematodes fed with ML1206 increased by 26% compared with the control group fed with OP50 under oxidative stress (Figure 6A,B). As a pressure-sensitive reporter gene, it is known that *hsp-16.2* enhances the protective effect of nematodes under stress injury [28]. In order to further investigate whether ML1206 influenced the expression of *hsp-16.2*, we used transgenic strain TJ375 to quantify the expression of HSP-16.2::GFP. In the absence of heat stress, the relative fluorescence intensity of HSP-16.2::GFP in nematodes fed with ML1206 increased by 53% compared with the control group fed with OP50. The relative fluorescence intensity of the nematodes fed with ML1206 increased by 59% compared with the control group fed with OP50 under heat stress (Figure 6C,D). The results suggested that ML1206 could increase the expression level of *sod-3* in the CF1553 strain and expression level of *hsp-16.2* in the TJ375 strain with or without environmental stress. 

### 2.9. Effect of ML1206 on the Lifespan of DAF-2 and DAF-16 Mutants

**Figure 7:**
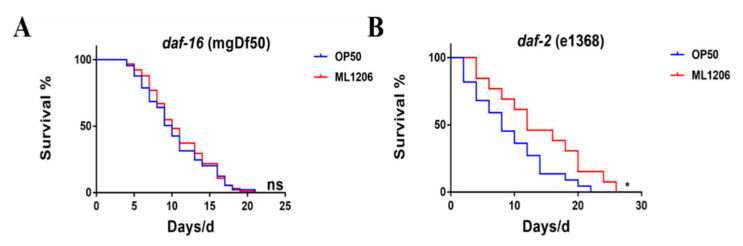
Effect of ML1206 on the lifespan of DAF-16 and DAF-2 mutants: (**A**) lifespan of GR1307 DAF-16 (mgf50) strain fed with ML1206 and OP50; and (**B**) lifespan of DR1572 DAF-2 (e1368) strain fed with ML1206 and OP50. The numbers of nematodes were calculated in Kaplan–Meier diagrams, and statistical significance was evaluated by the log-rank test. *p* < 0.05 is considered statistically significant. (*: *p* < 0.05; ns: *p* > 0.05).

To further explore the role of DAF-2 and DAF-16 in ML1206 mediated longevity effect, we conducted a study to determine the effect of ML1206 on lifespan in DAF-16 (mgDf50) mutant GR1307 and DAF-2 (e1368 III) mutant DR1572. Compared with the control group, the results indicated that ML1206 extended the lifespan of mutant DR1572 by 50.9% from 9.18 ± 1.28 days to 13.85 ± 2.05 days, but the mean lifespan of mutant GR1307 was 10.45 ± 0.43 days, and ML1206 did not prolong the survival time (Figure 7A,B, Table 1). The findings suggest that DAF-16 is essential for ML1206 to mediate the life extension effect, while DAF-*2* is not required.

## 3. Discussion

Aging is a complex process, accompanied by a progressive decline of physiological integrity, functional degradation, and increased vulnerability to common diseases [29]. In previous work, our findings have provided the first data to demonstrate that ML1206, as a probiotic, could enhance the anti-infection ability of *C. elegans* and prolong their lifespan [9]. Previous studies show that oxidative stress resistance is closely related to anti-aging ability. *Lactobacillus rhamnosus* CNCM I-3690 and *Weissella confusa* CGMCC 19,308 strains have shown that they could not only enhance resistance to stress but also could extend the lifespan of *C. elegans* [30,31]. In this study, the anti-aging and antioxidant ability and the exact mechanisms of ML1206 were described in *C. elegans*.

The direct way to explore antioxidant effects in organisms is to construct stress damage models induced by chemical or physical (thermal) stressors after treatment with antioxidants [16,32,33]. As shown in the results, the lifespan of wide-type *C. elegans* fed with ML1206 was prolonged by 28.7% under normal conditions, indicating a certain anti-aging effect. In addition, ML1206 significantly extended the mean lifespan of wide-type *C. elegans* by 48.8% under chronic oxidative stress. The results show that the antioxidant ability of ML1206 may be one of the reasons why it could prolong the lifespan of *C. elegans.* If the body is attacked under an environmental stress, such as ultraviolet rays, heat, and oxidative damage, more ROS are generated, resulting in a low survival rate [34,35]. As shown by the results (Figure 2), feeding either live or inactivated ML1206 increased the survival of *C. elegans* under oxidative stress and heat stress compared with the control fed with live or inactivated OP50, indicating that ML1206 was able to enhance the stress resistance of *C. elegans*, regardless of its living state. We found that ML1206 did not show significant antioxidant activity in vitro, but the analysis of the ML1206 genome showed that ML1206 may produce terpenoids highly similar to carotenoids (unpublished data). Shindo K et al. have found that glyco-C30-carotenoic acid produced by *Planococcus maritimus* showed potent antioxidant activity [36]. Thus, it is speculated that ML1206, whether live or inactivated, may produce some compounds or metabolites to enhance the stress resistance of nematodes and improve their survival rates. Within the antioxidant defense system of *C. elegans,* antioxidant enzymes such as CAT, GSH-PX and SOD are the main defenders, responsible for eliminating a large number of free radicals [37]. Compared with the control fed with OP50, the CAT of *C. elegans* fed with ML1206 was increased with or without oxidative stress and the GSH-PX was increased in *C. elegans* fed with ML1206 in the presence of oxidative stress, leading to the enhancement of total antioxidant capacity of *C. elegans*. Furthermore, as the antioxidant ability was enhanced, the intracellular level of ROS was significantly attenuated under oxidative stress in *C. elegans* fed with ML1206. This phenomenon is also observed among other probiotics. *Lactobacillus plantarum* As21 can activate the activities of CAT, SOD, and GSH-PX of the host, which then reduces the level of ROS and enhances the stress resistance, thereby protecting nematodes [38]. Therefore, ML1206 may improve the antioxidant capacity of nematodes by activating the antioxidant defense system and scavenging ROS against harsh stress damage. 

Life extension and oxidative stress resistance is usually attributed to the activation of certain signaling pathways, especially the *IIS* pathway [39,40]. The key antioxidant-related genes regulated by Insulin/IGF-1 signaling (*IIS* pathway), such as DAF-16, DAF*-2*, *sod-3*, *ctl-2* and *hsp-16.2*, have been widely concerned because of their important roles in regulating oxidative stress and affecting lifespan [41,42,43]. DAF-16, an ortholog of the FOXO transcription factor, could regulate the downstream target genes, such as *sod-3* encoding SOD [44], *ctl-2* encoding CAT [43] and *hsp-16.2* encoding heat shock protein [28]. *Bifidobacterium longum* enhanced tolerance to H_2_O_2_-induced stress and prolonged the lifespan in *C. elegans*, which were mediated by DAF-16 activation [45]. Zhang et al. have reported that oleanolic acid could increase the antioxidant capacity and prolong lifespan by upregulating the expression of DAF-16 target genes such as *sod-3*, *ctl-2*, and *hsp-16.2* [46]. Therefore, we explored DAF-16′s role in ML1206 mediated antioxidant effect of *C. elegans*, and its contribution to the downstream regulation of target genes *sod-3*, *ctl-2* and *hsp-16.2*. Our results showed that the localization percentage of DAF-16 transcription factor within the nucleus in *C. elegans* was markedly increased and the mRNA transcription of DAF-16 gene was activated with the treatment of ML1206. In this case, the greater presence of DAF-16 in the nucleus of *C. elegans* fed with ML1206 further upregulated the mRNA transcription of downstream target genes *sod-3*, *ctl-2* and *hsp-16.2*. Subsequently, the transgenic strains CF1553 and TJ375 were used to analyse the level of SOD-3::GFP and HSP-16.2::GFP in *C. elegans* treated with ML1206. It was found that feeding ML1206 increased the expression level of SOD-3::GFP in *C. elegans* more than the control group fed with OP50 with or without oxidative stress. The higher level of HSP-16.2::GFP of nematodes fed with ML1206 was also obversed than in the control group fed with OP50 with or without thermal stress. This finding was consistent with the previous results of an increased mRNA transcription level of genes *sod-3* and *hsp-16.2* obtained from RT-qPCR. In brief, feeding ML1206 activated transcription of DAF-16 and increased the percentage of DAF-16 localization in the nucleus, which further upregulated downstream antioxidant genes such as *sod-3*, *ctl-2* and *hsp-16.2*, and activated the antioxidant defense system in *C. elegans*; thus, the antioxidant capacity of *C. elegans* was significantly enhanced.

DAF*-2*, the insulin/insulin-like growth factor receptor homolog, is located upstream of DAF-16 and regulates the transcriptional activity of DAF-16 [47]. Above, we discussed how DAF-16 and the coded DAF-16 were impacted by ML1206, and its further upregulation of downstream target genes. Here, we will discuss the DAF-2 as a known upstream regulator of DAF-16, and whether it was one of the pathways via which ML1206 could impact activities associated with DAF-16. The previous study clarified that activating DAF-16 or inhibiting DAF-2 is the key to keep longevity [48]. The results obtained by RT-qPCR showed that the expression of DAF-2 was not significantly different between the *C. elegans* fed with ML1206 and OP50. Table 1 shows that the lifespan of DAF-2 (e1368) mutants fed with ML1206 was significantly prolonged, by 50.9%, than that of the control group. However, there was no significant difference in the lifespan of DAF-16 (mgDf50) mutants between the experimental and control groups. All the findings indicate that the lifespan extension effect of *C. elegans* fed with ML1206 is dependent on DAF-16, while DAF-2 might be nonessential to the longevity regulation mediated by ML1206. However, DAF-16 can not only be activated by DAF-2 in the *IIS* pathway, but also be activated by the AMP-activated protein kinase (AMPK) pathway, silent information regulator 2 (SIR-2.1) or germline signaling [49,50], to regulate the metabolism, strengthen stress resistance, and extend the lifespan of the nematodes [51,52]. It was reported previously that aspirin extended the lifespan of *C. elegans* by activating the AMPK pathway, which stimulates the expression of downstream target genes through a DAF-16 translocation-independent manner, rather than being activated by DAF-2 in *IIS* pathway [53]. SIR-2.1 has been reported to be able to extend the lifespan by interacting with 14-3-3 proteins and activating DAF-16 in *C. elegans* [54]. Huang et al. found that DhHP-6 increased the lifespan and resistance stress of *C. elegans*, which was dependent upon the enhancement of DAF-16 translocation from cytoplasm to nuclei, likely by activating the SIR-2.1/DAF-16 complex [55]. Thus, it is speculated that ML1206 may exert anti-aging and antioxidant effects on *C. elegans* by integrating different signal factors from other pathways related to DAF-16 instead of DAF-2 in *IIS* pathway, which requires further study. 

In conclusion, in this paper we used *C. elegans* to evaluate the antioxidant effect and life extension of ML1206 in vivo and reveal the underlying mechanism for the first time. As shown by the results, feeding ML1206 could enhance the stress tolerance of *C. elegans* and prolong the lifespan, with or without the presence of external stress. The antioxidant effect of ML1206 in *C. elegans* might be dependent on the FOXO/DAF-16 via activating the translocation of DAF-16 to the nucleus, thereby upregulating the mRNA level of antioxidant-related genes, such as *sod-3, ctl-2* and *hsp-16.2.* Furthermore, the activity of antioxidant enzymes was increased, and the accumulation of ROS was effectively reduced to defend against oxidative stress. Overall, the ML1206 strain acted as a promising potential probiotic with antioxidant ability in vivo that could be preferentially used in aquaculture to help defend against some diseases related to oxidative stress or slow down the speed of an organism’s caducity, with a view to producing good economic benefits.

## 4. Materials and Methods

### 4.1. Strains and Growth Condition

The ML1206 used in this study was isolated from the intestines of wild oysters (*Crassostrea gigas*) from the Yuanyao dock in Weihai City, Shandong Province. It was grown in marine agar 2216 (MA; Becton Dickinson, Franklin Lakes, NJ, USA) at 28 °C. *Escherichia coli* (*E. coli.* OP50), standard food for *C. elegans*, was grown in Luria-Bertani (LB) broth at 37 °C [56]. *C. elegans* strains used in this study were wild-type N2 Bristol, GR1307(mgDf50[DAF-16]), DR1572(e1368III[DAF-2]), SYD0716(muIs109[DAF-16p::GFP:: DAF-16cDNA+odr-1p::RFP]), TJ375(gpIs1[hsp-16.2p::GFP]) and CF1553(muIs84[(pAD76) sod-3p::GFP+rol-6(su1006)]), which were obtained from the Caenorhabditis Genetics Center (CGC, University of Minnesota, Minneapolis, MN, USA). All nematodes were grown and maintained at 20 °C on nematode growth medium (NGM) seeded with *E. coli* OP50 [56]. To prepare live bacteria lawns for *C. elegans* feeding, *E. coli* OP50 or ML1206 strain was harvested and washed three times in M9 buffer (Na_2_HPO_4_ 6 g, KH_2_PO_4_ 3 g, NaCl 5 g, MgSO_4_·7H_2_O 0.25 g in 1 L of distilled water and autoclaving), then was adjusted to a final concentration of 0.25 mg/µL in M9 buffer [9] and seeded on NGM (NaCl 3 g, Bacto peptone 2.5 g, 1 M potassium phosphate (pH 6.0) 25 mL, agar 17 g in 1 L of distilled water and autoclaving, then added cholesterol (5 mg/mL) 1 mL, 1 M CaCl_2_ 1 mL and 1 M MgSO_4_ 1 mL) plate mentioned above. All nematodes were synchronized for the experiment. The synchronization was carried out according to Herbenya’s method [57], and the eggs were incubated to the L1 stage at 20 °C and then were transferred to the NGM plate containing *E. coli* OP50 and grew to the L4 stage at 20 °C for the subsequent experiments.

### 4.2. Antioxidant Capacity of ML1206 In Vitro

The cultured OP50, ML1206, inactivated OP50, and inactivated ML1206 were centrifuged separately. The method of heat inactivation was as follows: the water bath was heated at 100 °C for 15 min [58]. The bacteria were washed with PBS buffer three times and ultrasonic crushed in the ice-water mixed bath (ultrasonic time 15 min, 5 S on, 10 S off, ultrasonic power 20 W). The supernatant was taken after centrifugation for testing. The T-AOC of ML1206 was determined by the total antioxidant capacity detection kit (Beyotime, Shanghai, China) according to the Ferric Reducing Antioxidant Power (FRAP) method. The T-AOC was represented as FeSO_4_ standard solution concentration/protein concentration. Protein concentration was measured using a BCA kit (Beyotime, Shanghai, China) to homogenize the results.

### 4.3. Oxidative Stress Resistance Assay

Synchronized to L4 stage, nematodes were pre-treated with 0.25 mg/mL ML1206 or OP50. After being treated for 48 h, the nematodes were transferred to 96-well plates containing Paraquat (100 mM) [59] and H_2_O_2_ (5 mM) [60] and then incubated for 6 h and 2 h [61], respectively. 60 nematodes were evenly divided into three plates each group. The survival rate of the N2 nematodes was calculated. If they failed to respond to a gentle touch with a platinum wire, nematodes were considered to be dead [9]. Survival rate = number of survivors/total number ×100%.

### 4.4. Heat Stress Assay

Synchronized to L4 stage, nematodes were incubated in the presence or absence of ML1206. After being treated for 48 h, nematodes were transferred to fresh NGM plates without foods. 60 nematodes were evenly divided into three plates each group. After being exposed 2 h at 37 °C and recovering for 2–3 h at 20 °C [62], the survival rate was scored. If they failed to respond to a gentle touch with a platinum wire, nematodes were considered to be dead. Survival rate = number of survivors/total number ×100%.

### 4.5. Lifespan Assay

To explore the effect of ML1206 on the lifespan of *C. elegans*, synchronized the wild-type strain (N2) and the mutant strains GR1307 DAF-16 (mgDf50) and DR1572 DAF-2 (e1368 III), and nematodes were grown at 20 °C until L4 stage. The nematodes were transferred to NGM plates with 0.1 mg/mL 5-FUDR and treated with OP50 or ML1206. In addition, to assess the lifespan of *C. elegans* under oxidative stress, the nematodes were covered with H_2_O_2_, a pro-oxidant stressor analysis of oxidative stress resistance in nematodes. The plates were incubated at 20 °C, and the numbers of live and dead nematodes were scored every two days. At the same time, they were shifted to fresh NGM plates every 2–3 days. A nematode was considered as dead if it failed to respond to a platinum wire. 

### 4.6. Determination of Antioxidant Enzyme Activity 

The antioxidant activity of ML1206 was measured in the presence and absence of oxidative stress in *C. elegans*. Synchronized to the L4 stage, nematodes were fed with ML1206 or *E. coli* OP50. After 48 h incubation, the nematodes were treated with or without 5 mM H_2_O_2_ for 2 h and washed with M9 buffer. Next, the nematodes were shaken for half an hour, washed three times to eliminate the influence of bacteria itself as much as possible, and centrifuged slightly. The nematodes were lysed using a rotor–stator on ice, accompanied by intermittent grinding (grinding 15 s, stopping 5 s) to prevent enzyme inactivation. The homogenate was centrifuged at 12,000 rpm, 4 °C for 3 min, and the supernatant was collected for enzymeatic activity determination. The T-AOC was determined using the Total Antioxidant Capacity Assay Kit (Beyotime, Shanghai, China), and each individual enzyme activity was determined using SOD, CAT, and GSH-PX activity kit (Nanjing JianCheng Bioengineering Institute, Nanjing, China) respectively. The protein content was determined by BCA Protein Assay Kit (Beyotime, Shanghai, China).

### 4.7. Analysis of Intracellular ROS 

The nematodes were treated and obtained according to the 4.6 method. The nematodes were transferred to a black 96-well plate containing a final concentration of 25 µM H_2_DCF-DA and incubated at 20 °C for 30 min in dark. The ROS in nematodes was detected and quantified by the Cell Imaging microplate detection system Cytation5 (Bio Tek, Winooski, VT, USA). The excitation wavelength was 485 nm, and the emission wavelength was 525 nm [63]. The measurement was performed every 20 min for a total of six measurements.

### 4.8. Real-Time Fluorescence Quantitative PCR Analysis

Synchronized to the L4 stage, nematodes were fed with ML1206 or *E. coli* OP50. After incubated for 48 h, the total RNA of nematodes was extracted using Illumina Truseq TMRNA spin Mini RNA Isolation Kit (GE Healthcare, Buckinghamshire, UK). cDNA was synthesized using ABscript II cDNA First Strand Synthesis Kit (ABclonal, Wuhan, China). qRT-PCR was carried out using 2X Universal SYBR Green Fast qPCR Mix (Abclonal, Wuhan, China) and ABI StepOne Plus Real-Time PCR detection system. In this study, *act-1* was chosen as the reference gene, and relative quantification of gene expression was performed using the 2^−ΔΔCt^ method [64]. All primers are shown in Table 2. Samples were repeated in triplicate.

### 4.9. Fluorescence Microscopy and Visualization Assay

The transgenic strains CF1553, TJ375, and SYD0716 were used to quantify the expression of stress-related genes *sod-3* and *hsp-16.2* and the nuclear translocation level of *DAF-16*. Synchronized to the L4 stage, nematodes were pre-treated with ML1206 or OP50 for 48 h. Next, the transgenic strains CF1553 and SYD0716 were exposed or not to 100 mM paraquat for 6 h to induce oxidative stress. In addition, the TJ375 strain was exposed or not to 37 °C for 2 h and recovered for 2–3 h at 20 °C. Subsequently, 10 nematodes in each experimental and control group were randomly selected to be anesthetized with 1mM hydrochloride levamisole [68] and were fixed on a 2% agarose pad. All the GFP fluorescence assays were performed with the filter (excitation wavelength 485 nm, emission wavelength 535 nm) and an Axio Scrope A1 fluorescence microscope (Carl Zeiss AG, Jena, Germany) connected to Zen Blue Software’s AxioCam 503 color digital camera for image acquisition and analysis, and the relative fluorescence intensity of the entire body was determined using ImageJ software.

### 4.10. Statistical Analysis

Every experiment was carried out in three biological and technical duplication. Three independent assays were carried out with each group. All data are represented as the mean ± SEM of three individual experiments. Statistical analysis was performed using GraphPad Prism version 7.0 (San Diego, CA, USA) and unpaired Student’s *t*-test to compare pairs of groups. Fluorescence intensity was quantified by ImageJ software. The statistical significance was determined *p* < 0.05.

## Figures and Tables

**Table 1:** The mean lifespans of three types of nematodes in the presence of ML1206.

Strains	Treatment	Mean Lifespan Days	Median	Significance
Wide type (normal condition)	OP50 ML1206	11.47 ± 1.18 14.76 ± 1.35	12 14	*
Wide type (with stress)	OP50 ML1206	4.63 ± 0.44 9.04 ± 0.61	3 5	***
GR1307 *DAF-16* (*mgDf50)*	OP50 ML1206	10.40 + 0.45 10.58 + 0.43	10 10	ns
DR1572 *DAF-2* (e1368)	OP50 ML1206	9.18 ± 1.28 13.85 ± 2.05	8 12	*

*: *p* < 0.05; ***: *p* < 0.001; ns: *p* > 0.05.

**Table 2:** The Primer sequences used for Real-time PCR detection.

Gene	Forward Primer	Reverse Primer
* *act-1* [9]	CCCCACTCAATCCAAAGGCT	GTACGTCCGGAAGCGTAGAG
*sod-3* [65]	GGCTAAGGATGGTGGAGAAC	ACAGGTGGCGATCTTCAAG
*ctl-2* [66]	GAGAATGTGCCAGAACTTTGC	CTTGACACGAGCTCCAAAATC
*skn-1* [65]	GACGTCAATTTATGGAGTGTCG	GAAGATGTTTTGTCGTGATCCG
*DAF-2* [66]	GGATAAAGGCGAATCAAAGTGTC	CGATACACTTTCCCTTGTGATAGAC
*DAF-16* [65]	TCAAGCCAATGCCACTACC	TGGAAGAGCCGATGAAGAAG
*hsp-16.2* [67]	TATGGCTCTGATGGAACG	GATTGATAGCGTACGACC

* *act-1* is an internal reference gene.

## Data Availability

The data presented in this study are available in the main text and the supplementary materials of this article.

## Supplementary Materials

The following are available online at https://www.mdpi.com/article/10.3390/md21010001/s1. Figure S1: Effect of ML1206 strain on resistance to UV stress in *C. elegans*; Figure S2: The total antioxidant capacity of ML1206 strain; Figure S3: The DPPH free radical scavenging ability of ML1206 strain [19,69,70,71].
