# Supplementary material for: Planococcus maritimu ML1206 Strain Enhances Stress Resistance and Extends the Lifespan in Caenorhabditis elegans via FOXO/DAF-16
Source: Mar Drugs. 2022 Dec 20;21(1):1. doi: 10.3390/md21010001 (PMC9866299; doi:10.3390/md21010001)
Supplement: Supplementary file 1 [file marinedrugs-21-00001-s001.zip › marinedrugs-2052031-supplementary.pdf]

Supplemental data

Methods

1. ***UV stress resistance assay***

The nematodes were synchronized until L4 stage and pre-treated with 0.25 mg/mL ML1206 or *E. coli* OP50. After incubated 48 h, the nematodes were transferred to fresh NGM plates without bacteria. 60 worms were evenly divided into three plates each group, covered a dose of 1,000 J/m^2 expose to 254 nm UV radiation for 2 h, and then recovered for 24 h at 20 ℃ [69], the survival rate was scored. If they fail to respond to a gentle touch with a platinum wire, worms were considered to be dead. The experiment was carried out for three times. Survival rate = number of survivors/total number ×100%

2. ***Ferric-reducing antioxidant power*** (FRAP) ***assay***

The cultured OP50, ML1206, inactivated OP50 and inactivated ML1206 were centrifuged separately. The method of heat inactivation was as follows: the water bath was heated at 100 ℃ for 15 min. The supernatant was discarded, washed with PBS buffer three times, and ultrasonic crushing was carried out in the ice-water mixed bath (ultrasonic time was 15 min, 5s on, 10s off, 20W). The supernatant was taken after centrifugation at 4 ℃ for testing. The total antioxidant capacity of ML1206 was determined by the total antioxidant capacity detection kit (Beyotime, Shanghai, China) according to the ferric-reducing antioxidant power (FRAP) method. For the FRAP method, the total antioxidant capacity (T-AOC) was expressed as $FeSO_4$ standard solution concentration/protein concentration. Protein concentration was measured using a BCA kit (Beyotime, Shanghai, China) to homogenize the results. The experiment was repeated for three times, and the results were averaged.

3. ***2,2-diphenyl-1-picryhydrazyl radical (DPPH) free radical scavenging assay***

The cultured OP50 and ML1206 were centrifuged respectively and divided into two groups, one of which was exposed to 5mM H_2O_2 for 2 h. The supernatant was discarded, washed with PBS buffer three times, and ultrasonic crushing was carried out in the ice-water mixed bath (ultrasonic time was 15 min, 5s on, 10s off, 20W). The supernatant was taken after centrifugation at 4 ℃ for testing. To determine DPPH radical scavenging activity of the samples, 100 μL supernatant and 100μL DPPH ethanol solution were mixed. The mixture was then incubated in dark at 37 ℃ for 30 min. The absorbance of the mixture was measured at a wavelength of 517 nm. DPPH radical scavenging activity (%)=[(Absorbance $_{blank}$−Absorbance $_{sample}$)/Absorbance $_{blank}$]×100%. Protein concentration was measured using a BCA kit (Beyotime, Shanghai, China) to homogenize the results. The experiment was repeated for three times, and the results were averaged.

Results

1. ***Effect of ML1206 on the UV stress tolerance of C. elegans***

Ultraviolet (UV) is a kind of ubiquitous stress in the external environment, and it is a fairly typical DNA damaging agent[70]. In addition, absorption of ultraviolet energy can produce many pairs of energy through energy exchange reactions[71]. In order to evaluate whether ML1206 have a significant protection effect on nematodes under UV stress, the survival of

nematodes was measured after exposed for 2 h (Figure S1). The results showed that there was no significant difference between the groups fed with ML1206 or OP50 in the presence of UV stress.

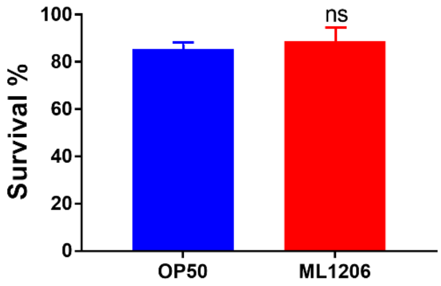

Figure S1. Effect of ML1206 on resistance to UV stress in *C. elegans*.

Note: Survival of the worms were determined after exposed for 2 h. Results were represented as mean ± SEM of three independent experiments and were statistically analyzed by an unpaired Student's t-test. (ns: $p > 0.05$)

2. *The T-AOC of ML1206 in vitro*

The antioxidant capacity of ML1206 *in vitro* was evaluated by FRAP method. The results showed that the T-AOC of OP50 was 0.3188, and the T-AOC of ML1206 was 0.3043. There was no significant difference between the two groups. In addition, the antioxidant capacity of inactivated OP50 and ML1206 were tested. The T-AOC of inactivated OP50 was 0.5153, and the relative T-AOC of inactivated ML1206 was 0.6043. There was also no significant difference between them. As shown in Figure S2, the ML1206 strain has no significant T-AOC in *vitro*.

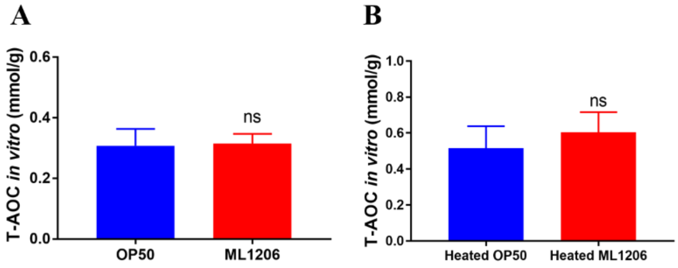

Figure S2. The relative total antioxidant capacity of ML1206 *in vitro*. (A)The T-AOC of ML1206 *in vitro*. (B) The T-AOC of ML1206 *in vitro*. Notes: Results are represented as mean ± SEM of three independent experiments and were statistically evaluated by an unpaired Student's t-test. (ns: $p > 0.05$)

3. *The DPPH free radical scavenging activity of ML1206*

It has been found that the most common and reliable methods are the DPPH methods when determining the antioxidant activity and scavenging capacity against ROS [19]. DPPH radical scavenging Rate determination results of OP50 and ML1206 as shown in Figure S3. Whether none oxidative stress condition or oxidative stress condition, ML1206 and OP50 both showed similar DPPH radical scavenging activity. There was no significant difference between them. These results indicate that ML1206 and OP50 both have no obvious ability to scavenge free radicals *in vitro*.

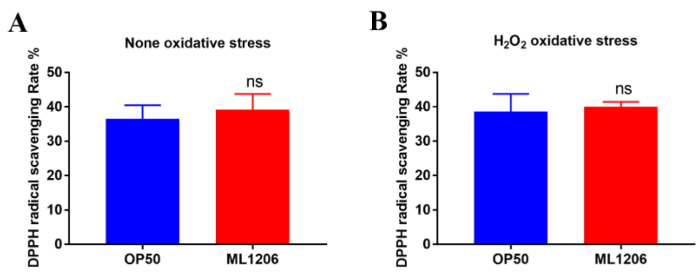

Figure S3. The DPPH free radical scavenging ability of ML1206 strain. (A) The DPPH free radical scavenging ability of ML1206 under none oxidative stress condition. (B) The DPPH free radical scavenging ability of ML1206 under oxidative stress condition. Results are represented as mean ± SEM of three independent experiments and were statistically evaluated by an unpaired Student's t-test. (ns: P>0.05)
